# Burden of disease and economic impact of human *Streptococcus suis* infection in Viet Nam

**DOI:** 10.1093/trstmh/trz004

**Published:** 2019-02-27

**Authors:** Vu Thi Lan Huong, Hugo C Turner, Nguyen Van Kinh, Pham Quang Thai, Ngo Thi Hoa, Peter Horby, H Rogier van Doorn, Heiman F L Wertheim

**Affiliations:** 1 Wellcome Trust Asia Programme—Oxford University Clinical Research Unit, 78 Giai Phong, Dong Da, Ha Noi, Viet Nam; 2 Wellcome Trust Asia Programme—Oxford University Clinical Research Unit, 764 Vo Van Kiet, Ward 1, District 5, Ho Chi Minh, Viet Nam; 3 Centre for Tropical Medicine and Global Health, Nuffield Department of Medicine, University of Oxford, Old Road campus, Roosevelt Drive, Headington, Oxford, UK; 4 National Hospital for Tropical Diseases, 78 Giai Phong, Dong Da, Hanoi, Viet Nam; 5 National Institute for Hygiene and Epidemiology, 131 Lo Duc, Hai Ba Trung, Hanoi, Viet Nam; 6 Radboud UMC, Geert Grooteplein Zuid 10, GA, Nijmegen, the Netherlands

**Keywords:** burden of disease, cost of illness, incidence, meningitis, *Streptococcus suis*, zoonosis

## Abstract

**Background:**

*Streptococcus suis* is a zoonotic disease mainly affecting men of working age and can result in death or long-term sequelae, including severe hearing loss and vestibular dysfunction. We aimed to quantify the burden of disease and economic impact of this infection in Viet Nam.

**Methods:**

The annual disease incidence for the period 2011–2014 was estimated based on surveillance data using a multiple imputation approach. We calculated disease burden in disability-adjusted life years (DALYs) and economic costs using an incidence-based approach from a patient’s perspective and including direct and indirect impacts of *S. suis* infection and its long-term sequelae.

**Results:**

The estimated annual incidence rate was 0.318, 0.324, 0.255 and 0.249 cases per 100 000 population in 2011, 2012, 2013 and 2014, respectively. The corresponding DALYs lost were 1832, 1866, 1467 and 1437. The mean direct cost per episode was US$1635 (95% confidence interval 1352–1923). The annual direct cost was US$370 000–500 000 and the indirect cost was US$2.27–2.88 million in this time period.

**Conclusions:**

This study showed a large disease burden and high economic impact of *S. suis* infection and provides important data for disease monitoring and control.

## Introduction

*Streptococcus suis* is a bacterium that causes substantial losses in the pig industry worldwide and is a common cause of zoonotic bacterial meningitis, mainly affecting working-age men in pig-rearing areas, particularly in Southeast Asia.^1,2^ The number of reported cases rapidly increased to >1600 cases in a 2013 review of publications.^3^ More than 500 cases had been reported from Viet Nam in research settings in some national and provincial hospitals by 2015.^4–8^ These reports include only cases that were identified and reported, likely representing only a small fraction of the real burden. The most common clinical presentation of human *S. suis* infection is purulent meningitis, severe sepsis, endocarditis, endophthalmitis and arthritis.^2^ In addition to substantial mortality (13% on average, 0–6% in Viet Nam), important sequelae include severe to complete sensineural hearing loss with or without vestibular dysfunction.^2^

Despite the proliferation of data on *S. suis* infection and increased awareness within scientific and medical disciplines of this important infection, there has been no reduction in incidence, particularly in Asian countries.^3^ This may be explained by the lack of awareness among community members and policymakers of the impact of the disease in terms of both health and economic consequences caused by premature deaths and long-term disability. To our knowledge, there have not been any research attempts to quantify the health and economic burden of *S. suis* infection in humans globally. Underutilization or lack of microbiology capacity in local hospitals, the use of antibiotics prior to hospital admission and sampling and poor continuity of care present a real challenge in attempts to quantify the burden of disease in resource-restricted settings such as Viet Nam. This study aimed to support policymakers in prioritizing and justifying resource allocation for the prevention and control of *S. suis* by quantifying the annual incidence, the disease burden in disability-adjusted life years (DALYs) and the economic impact of human *S. suis* infection in Viet Nam.

## Materials and methods

We estimated the burden of disease and economic impact of human *S. suis* infection following an incidence-based approach: all health and economic consequences attributable to the infection were captured through an outcome tree describing the causal relationships between these consequences and the initial infection. The impact of all consequences was attributed to one point in time when the infection occurred.^9^ The annual incidence of disease was estimated using a model-based approach with input data from the national surveillance of notifiable diseases for the years 2011–2014. Data on mortality and disability were obtained through a systematic literature review^2^ and a prospective follow-up study of patients with laboratory-confirmed *S. suis* infection admitted to the National Hospital for Tropical Diseases (NHTD) in Hanoi, Viet Nam between November 2014 and October 2015.^10^

### Annual incidence

**Figure 1.**
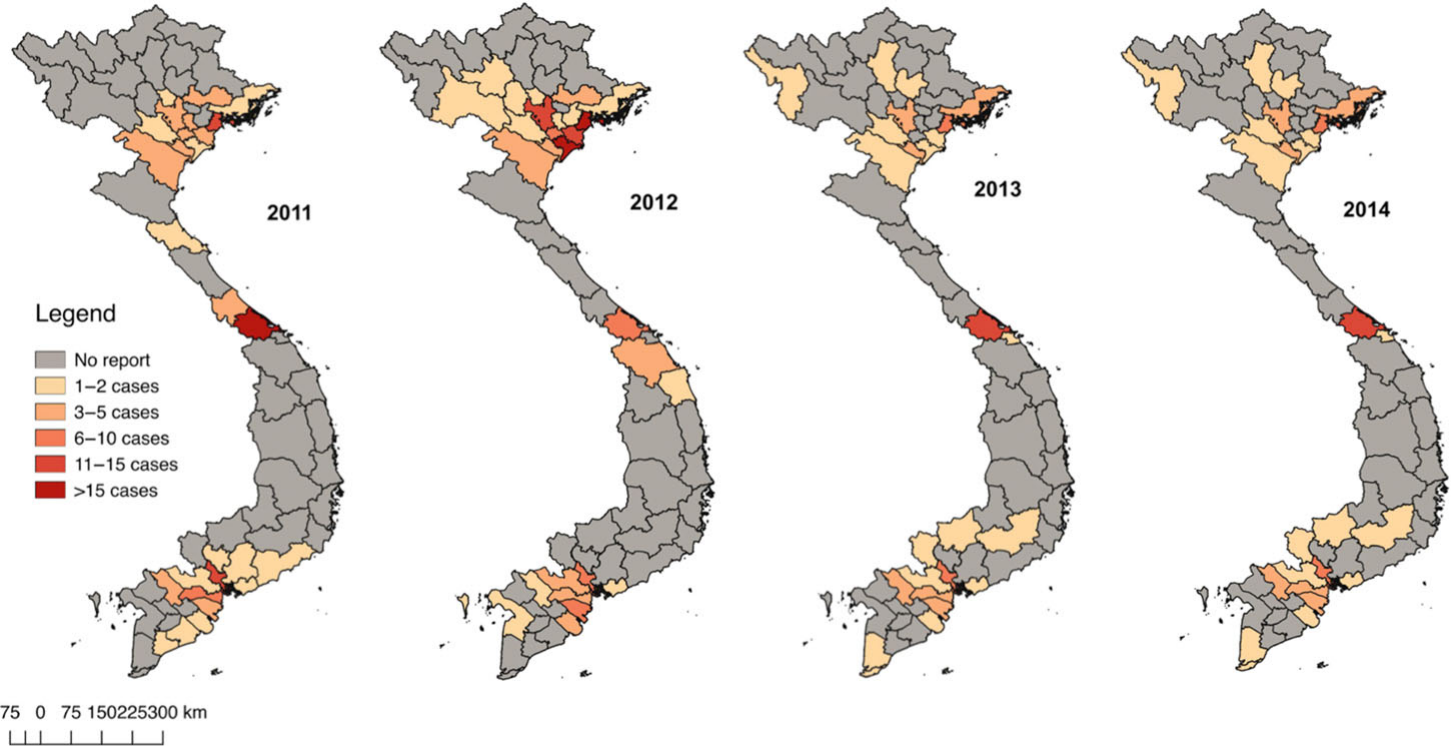
Distribution of *S. suis* cases reported to the national surveillance system by province in Viet Nam in 2011–2014.

*S. suis* has been a notifiable communicable disease in Viet Nam since 2011. Health facilities are required to report cases of culture confirmed *S. suis* infection within 24 h of identification to the local health preventive unit. However, many provinces do not report any *S. suis* cases, as shown in their reports for the period 2011–2014 (Figure 1). It is likely that this is due to underdiagnosis and/or underreporting, because pig farming is a common practice and pork is consumed by >98% of households in Viet Nam and in research studies *S. suis* is the most frequent causative agent identified in adult meningitis.^11^ Incidence data from non-reporting provinces were therefore treated as missing data in this analysis and imputed using a systematic and valid approach as described below.

The incidence was modelled using a set of predictor variables for which data were available at the provincial level from the national General Statistics Office of Viet Nam (http://www.gso.gov.vn). These variables were selected based on their possible association with disease incidence or with the probability of data missingness, including geographic region, human population density, pig density, proportion of rural population, number of hospitals, income, immigration rate, outmigration rate and proportion of skilled workers (see Supplementary Table 1). We used a multiple imputation approach, which is fundamentally a repeated imputation process taking *m* draws from a posterior predictive distribution for the missing data under a specific imputation model.^12^ All nine predictors were included in the imputation model, which generates *m*=100 imputed datasets with 10 iterations for each imputation cycle. The total disease incidence was calculated for each imputed dataset separately and the results were pooled across 100 imputed datasets to produce the mean incidence with a variance that took into account both within-dataset and between-dataset variation (more details in the Supplementary Data).

### Disease outcome tree

**Figure 2.**
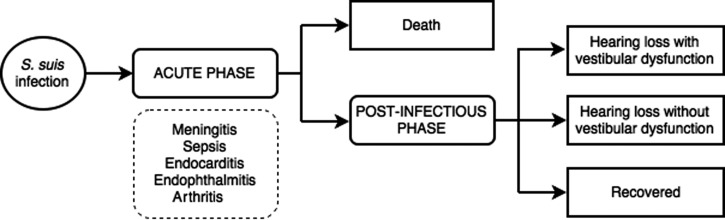
Outcome tree for *S. suis* infection in humans summarizing different states from acute phase to clinical outcomes of the disease.

We constructed an operational outcome tree (Figure 2) describing the main clinical states and outcomes resulting in morbidity/mortality. The acute phase represents all possible clinical presentations including meningitis during hospitalization, while the post-infectious phase includes the transitory states that patients surviving *S. suis* infection might go through after hospital discharge. The two main long-term disease sequelae (hearing loss and vestibular dysfunction) were captured in this outcome tree. These can occur in patients with and without meningitis, although the rate of hearing loss is higher in meningitis compared with non-meningitis cases. Hearing impairment can occur alone or accompanied by vestibular dysfunction. Vestibular dysfunction without hearing loss is uncommon in *S. suis* infection, therefore this outcome was excluded from the outcome tree. Outcomes affecting organs other than the hearing and vestibular system (e.g. vision loss) may occur, however, such outcomes are uncommon and data are insufficient to quantify the incidence of these outcomes from both the available literature and our follow-up study.

A gamma distribution was assumed for the incidence of disease and subsequent health states, with shape and scale parameters derived from the estimated mean and uncertainty interval. Transitioning probabilities (fixed values) and the durations of different health states (average and range) were derived using Vietnamese data if a reasonable estimate was available. Otherwise, estimates were obtained from the literature through the systematic review (Table 1). The numbers of cases in each state were estimated from the simulated annual incidence using probabilities of progressing from one state to another. We applied a case fatality rate (CFR) of 12.8%, which was the pooled estimate in the meta-analysis of all large studies.^2^ However, we also checked how the burden estimates vary if applying a smaller CFR of 4%, the pooled estimate for studies with meningitis patients. In the follow-up study, all cases with severe hearing loss showed vestibular dysfunction,^10^ therefore we did not consider the impact of severe hearing loss without vestibular dysfunction in *S. suis* survivors. Hearing loss was defined based on audiometry, which measured the average of hearing thresholds over four frequencies (0.5, 1, 2 and 4 kHz) in the better ear.^10^ We categorized hearing as normal (if the average was <20 dB), mild (20–34 dB), moderate (35–49 dB), severe loss (50–79 dB) and profound to complete loss (≥80–94 dB).

**Table 1.** Summary of parameters for estimating disease burden and economic impact of human *S. suis* infection in Viet Nam

Parameter	Value	Distribution	Source
Annual disease incidence (per 100 000 population)	Mean (95% UI)	Gamma	Simulated from surveillance data
2011	0.318 (0.306–0.331)		
2012	0.324 (0.313–0.335)		
2013	0.255 (0.250–0.261)		
2014	0.249 (0.238–0.261)		
Transition rate (%)	Mean		
Case fatality rate	12.8	Fixed	Systematic review^2^
Hearing loss + VD		Fixed	Follow-up study^10^
Mild	0.266		
Moderate	0.151		
Severe	0.111		
Profound/complete	0.288		
Hearing loss without VD^a^		Fixed	Follow-up study^10^
Mild	0.134		
Moderate	0.027		
Duration (days)	Mode (min, max)	Beta-pert	
Acute infectious episode	17.4 (13, 19.2)		Systematic review^2^
Post-infectious phase^b^	90 (15, 270)		Follow-up study^10^
Disability weight	Mean (95% UI)	Beta	GBD 2013^13^
Acute episode	0.133 (0.088–0.190)		
Post-infectious phase	0.219 (0.148–0.308)		
Hearing loss + VD^c^			
Mild	0.122 (0.083–0.166)		
Moderate	0.137 (0.099–0.181)		
Severe	0.253 (0.220–0.291)		
Profound/complete	0.294 (0.263–0.330)		
Hearing loss without VD			
Mild	0.01 (0.004–0.019)		
Moderate	0.027 (0.015–0.042)		
Age of onset of acute episode (years)	49.18	Fixed	Case series^d^

UI: uncertainty interval; VD: vestibular dysfunction; GBD: Global Burden of Disease.

^a^All cases with severe to profound/complete hearing loss showed vestibular dysfunction as observed in the follow-up study.

^b^Post-infectious phase occurs after the acute phase of an infectious disease, including consequences such as fatigue, emotional lability and insomnia. The lay description used in the Global Burden of Diseases 2013 was the person ‘is always tired and easily upset. The person feels pain all over the body and is depressed’.

^c^This was derived using a multiplicative method combining both the states of hearing loss and the state of vestibular dysfunction (see Supplementary Data).

^d^Age of disease onset was estimated from a case series from 2011 to 2015 at the NHTD. Specific mean age at onset was calculated for all age groups by sex for DALY calculations.

### Burden of disease in DALYs

The DALY measure is the summation of the number of years of life lost due to premature mortality (YLL) and the number of years of healthy life lost due to disability (YLD) (Supplementary Data). A disability weight between 0 (perfect health) and 1 (death) is used to adjust the years lived with disability in the YLD measure for each health state. Neither age weighting nor discounting was applied following the current practice in analysing global burden of diseases.^14^ We applied the Viet Nam life table for 2009^15^ to calculate the remaining life expectancy for each sex and age group. Disability weights were taken from the Global Burden of Diseases study for the year 2013^13^ (Table 1). Estimates were presented for the following age groups by sex: 15–44 y, 45–59 y and ≥60 y (*S. suis* infection has not been reported in children in Viet Nam). Uncertainty was determined through Monte Carlo simulations by iteratively simulating random values for each parameter according to its predefined distribution. Sensitivity analysis was performed using linear regression of the overall DALY estimates against the simulated values for the input parameters to identify parameters that cause significant uncertainty in the overall DALY estimates.

### Cost of illness

We quantified the economic impact using a cost-of-illness approach from a patient’s perspective. Costs generated throughout the lifetime of an incident *S. suis* case were summed to obtain the total economic impact incurred. These included direct medical costs (costs for any health care services for disease detection, treatment, continuing care and rehabilitation related to the infection episode or associated sequelae), direct non-medical costs (costs for travelling, food, accommodation and any other costs related to the infection episode and associated sequelae) and indirect costs (the value of the productivity losses incurred by the patients and their informal caregivers related to the infection episode and associated sequelae). The productivity costs incurred by the patients’ informal caregivers were included within the indirect costs and not the direct non-medical costs (because in Viet Nam family members usually stayed in the hospital to provide care to the patient).

Primary cost data were collected as part of the follow-up prospective study at the NHTD.^10^ The NHTD is a public hospital receiving government funding for overhead costs, including labour costs and assets. Operational costs are covered by health insurance and patient out-of-pocket payments. The insurance coverage rate in Viet Nam is not high among near-poor people (people having an income up to 1.5 times the poverty threshold): 26% compared with 63% for the national average coverage.^16^

Forty-seven patients with laboratory-confirmed *S. suis* infection between November 2014 and October 2015 at the NHTD were interviewed at hospital discharge and at 3 months and 9 months after discharge. Cost data were collected from hospital charts (formal hospital fees, insurance coverage) and interviews of patients and their relatives. Data collected from interviews included any informal fees and other medical and drug costs paid outside of standard hospital financial regulations of the NHTD and non-medical costs arising from hospitalization, such as food, transport and accommodations. For indirect costs, we asked the respondents about any income loss due to sickness and caregiving activities for both patients and caregivers.

We estimated indirect costs (productivity loss) due to premature mortality and long-term disability as the product of time loss and average potential earnings, taking a human capital approach (details on cost calculations are provided in the Supplementary Data).

To have a broader picture of the economic burden, we also interviewed about other impacts, including the patient’s time (number of hours per week) spent on paid and unpaid work before and after illness, the patient’s income earned before and after illness and any consequences caused by the illness and its sequelae. Unpaid work was defined as activities that the person spent time on but does not get paid for, such as household chores, shopping, childcare and voluntary work. However, these were not included in the cost of illness calculations.

We performed a one-way sensitivity analysis to assess the impact of uncertainty in each of the input parameters on the total cost of disease. We varied one parameter at a time while fixing the other parameters at their expected values.

Costs were collected and estimated at the prices of 2014–2015 (during the time of the follow-up study) in Vietnamese dong (VND). The impact of inflation during this time period was not significant and therefore was not adjusted for. We summarized the costs as mean, median, minimum and maximum in US dollars; 1 million VND was equivalent to US$46.38 at the average exchange rate between January 2014 and December 2015 (http://usd.fx-exchange.com/vnd/exchange-rates-history.html). These costs were used to estimate the total cost of illness for the years 2011–2014 (all expressed in 2014–2015 prices).

A 3% discount rate was applied to costs relating to long-term sequelae and premature mortality occurring after 2014.^17,18^ Differences in costs were tested using the Welch two-sample t-test, with unequal variances on a log scale. We used log-binomial models to calculate age-adjusted prevalence ratios for being unemployed between patients with and without long-term sequelae. All data processing and analysis was performed using R software (version 3.2.2; R Project for Statistical Computing, Vienna, Austria).

## Results

### Annual incidence

The annual incidence was estimated at 0.318, 0.324, 0.255 and 0.249 cases per 100 000 population in 2011, 2012, 2013 and 2014, respectively. Assessment of convergence and distributions of residuals showed the imputation was appropriate and valid data have been imputed to the overall incidence (Supplementary Figure 1 and 2). The estimated incidence rates were about two to three times the rate directly calculated from the number of cases reported to the surveillance system (0.08–0.15 cases per 100 000 population).

#### DALYs

**Figure 3.**
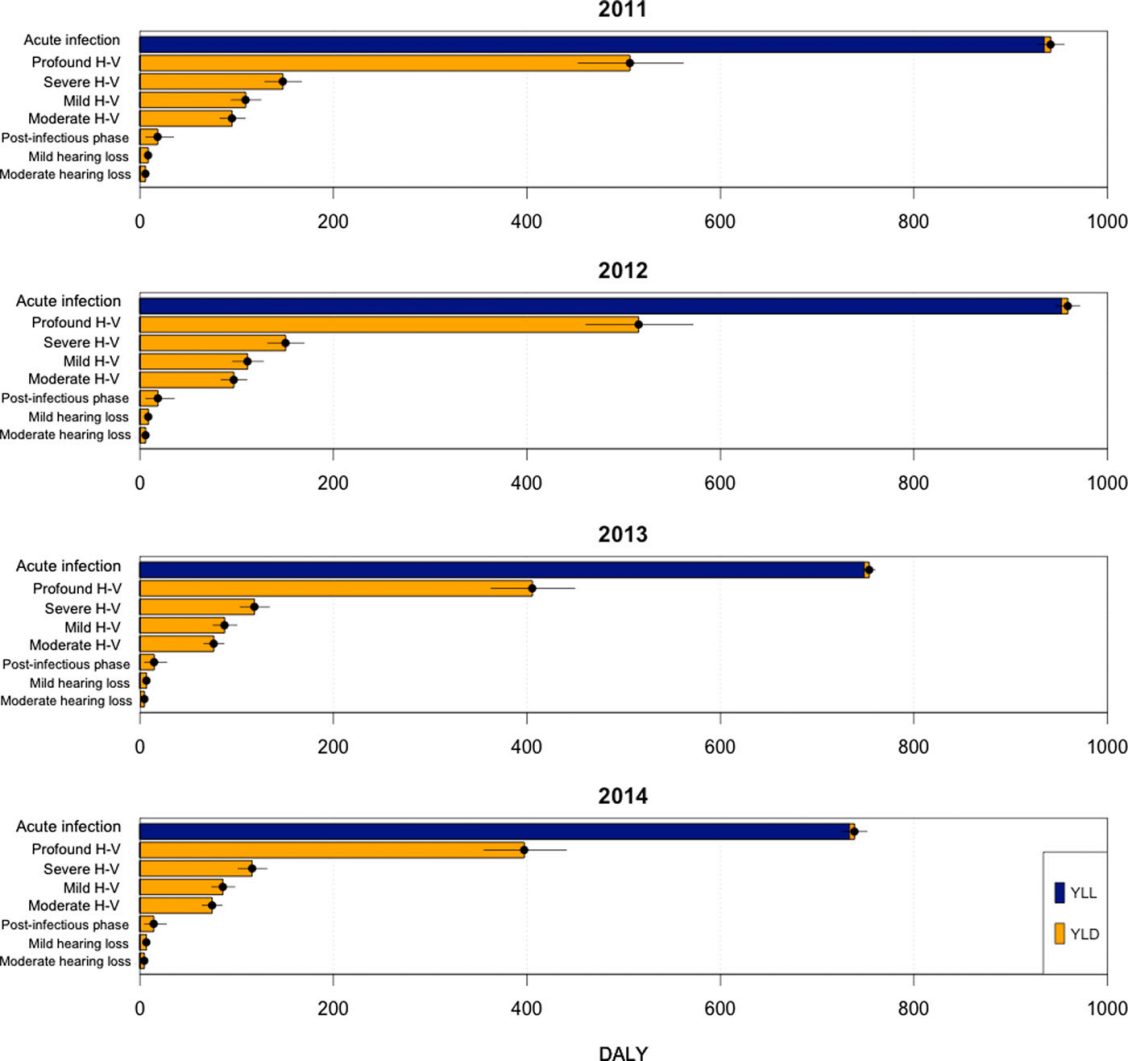
Estimated annual DALY burden caused by acute infection, post-infectious phase and long-term sequelae (hearing loss with vestibular dysfunction [H-V] and hearing loss without vestibular dysfunction) of human *S. suis* infection in Viet Nam in 2011–2014. Dark blue bar represents YLLs due to premature mortality; yellow bar represents YLDs.

The estimated disease burden of human *S. suis* infection in Viet Nam was 1832, 1866, 1467 and 1401 DALYs lost in the years 2011, 2012, 2013 and 2014, respectively (Table 2). The DALYs burden was disproportionately distributed towards men and people of working age. Overall, deaths due to acute infection account for the largest proportion of DALYs lost each year (>50%) when using the CFR of 12.8% (Figure 3). If we used a low CFR of 4%, the number of DALYs was reduced to 1189, 1212, 952 and 933, respectively, with 25% due to premature deaths.

**Table 2.** Annual total disease burden and economic impact of human *S. suis* infection in Viet Nam

Year	DALY^a^ (overall)	DALY by age group			DALY by sex		Direct costs^b^ (thousand $US)	Indirect costs^b^ (thousand $US)	
		15–44 y	45–59 y	≥60 y	Male	Female		Mortality	Morbidity
2011	1832	887	835	110	1584	248	499	1225	1576
	(1777–1888)						(142–1204)	(1176–1274)	(1513–1638)
2012	1866	904	850	112	1613	253	499	1260	1620
	(1811–1923)						(143–1195)	(1218–1302)	(1567–1674)
2013	1467	710	669	88	1267	200	386	1004	1291
	(1425–1511)						(112–914)	(982–1025)	(1264–1319)
2014	1401	680	638	83	1212	189	370	991	1275
	(1357–1446)						(105–897)	(946–1036)	(1216–1333)

^a^DALYs are presented as means; those in brackets are the 95% credibility intervals calculated through Monte Carlo simulations.

^b^Costs are presented as means; those in brackets are the lower and upper bound based on the 95% uncertainty interval of the estimated annual incidence generated through the imputation process. Primary direct costs were collected in 2014–2015.

Disability due to profound or complete hearing loss with vestibular dysfunction caused 506, 516, 406 and 397 DALYs in these years, accounting for more than half of the total YLD each year. The remaining disability was caused by other conditions, including acute illness, post-infectious period and mild to moderate hearing loss without vestibular involvement. Uncertainty in disability weight of profound or complete hearing loss had the greatest influence on the overall uncertainty in the estimated DALYs (Supplementary Figure 3).

### Cost of illness

The mean direct cost per *S. suis* infection episode was US$1635 (95% confidence interval [CI] 1352 to 1923), of which patients had to pay a mean medical cost of US$1046 and non-medical costs of US$589 (Table 3). Only half of the patients had insurance coverage (24/47), and among those insured, the coverage levels varied from 29% to 96% of the total hospital costs. Patients without health insurance had to pay about twice the medical costs of patients with insurance (US$1428 versus US$677, Welch t-test, p<0.001). Patients with long-term severe hearing loss paid significantly higher medical costs than those with non-severe impairment (US$765 versus US$505, p=0.03). No significant difference was observed in direct non-medical costs between groups by insurance and by hearing impairment severity.

**Table 3.** Direct costs per case of *S. suis* infection in patients hospitalized at the NHTD collected in 2014–2015 in Viet Nam

Type of direct costs	Mean ($US)	Median ($US)	Range ($US)
Medical costs (total cost^a^)	1431	1260	347–3094
Antibiotics	531	473	107–1448
Metabolites	292	356	97–422
Other drugs	94	62	24–498
Laboratory	226	225	59–381
Bed	147	90	19–1461
Services	41	12	3–304
Imaging	20	5	2–153
Consumables	34	34	12–90
Medical costs (paid by patient^b^)	1046	928	74–3094
Non-medical costs^c^	589	417	0–1855
Total direct costs^d^	1635	1375	473–4832

^a^Total medical costs (and the breakdown components underneath) included the out-of-pocket payments made by patients plus the payments covered by any health insurance scheme.

^b^These medical costs are the out-of-pocket payments made by the patients and excluded payments covered by health insurance.

^c^These included all direct non-medical costs paid by patients.

^d^Total direct costs included all direct medical and non-medical costs paid by patients, excluding payments made by health insurance.

**Figure 4.**
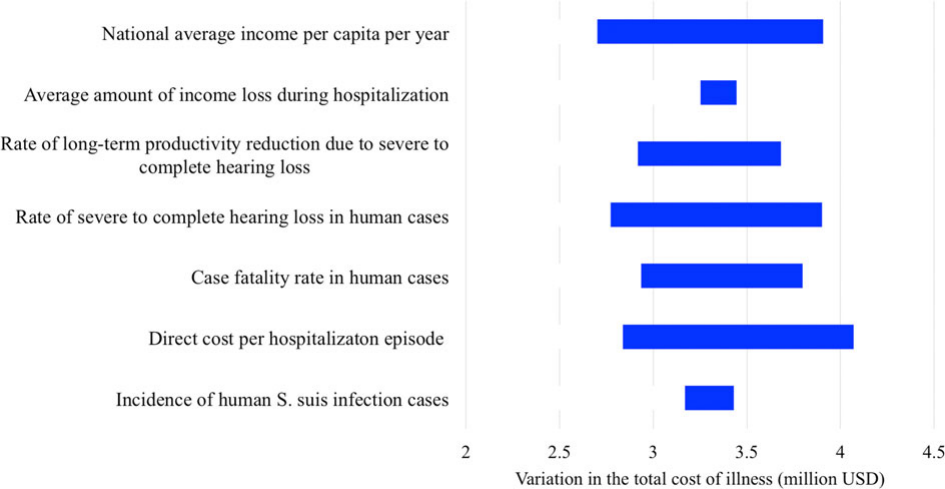
One-way sensitivity analysis showing the effect of changing the expected value in each parameter on the total cost of human *S. suis* infection in Viet Nam in 2011.

The annual direct cost was approximately US$370 000–500 000 between 2011 and 2014 in total (Table 2). The indirect cost was US$2 270 000–2 880 000, representing approximately 85% of the total cost. Sensitivity analysis showed that most of the variation in the total cost of illness was due to direct hospitalization costs, average national income level, rate of severe hearing loss in patients, case fatality rate and rate of productivity reduction in hearing impaired patients (Figure 4).

Twenty-three of 41 patients (56%) who had a paid occupation before hospitalization returned to their occupation by 9 months after hospital discharge (Table 4). Among these, eight had to change occupation due to health conditions (reduced hearing, dizziness, weakness), which prevented them from performing heavy duties such as construction work. The reported number of paid work hours per week was also significantly reduced (mean reduction of 13.6 h [95% CI 2.6 to 24.7], p=0.02, paired t-test). Patients who had moderate to complete hearing loss had a 2.6 times higher risk of unemployment at 9 months after discharge compared with other patients (64% versus 23%, prevalence ratio 2.6 [95% CI 1.2 to 7.9]).

**Table 4.** Impact of *S. suis* infection on work and income of infected patients hospitalized at the NHTD in 2014–2015 in Viet Nam

Variable	Summary values
Patients in paid work before illness	41/47 (87%)
Patients returning to paid work after 9 months since discharge^a^	23/41 (56%)
Among those with severe hearing loss	5/14 (36%)
Among those with non-severe hearing loss	16/25 (64%)
Impact of diseases on time for work (mean, median, minimum–maximum)	
Time between hospital discharge and returning to paid work^b^ (months)	3.2, 3, 0.5–9
Time spent on paid work per week before illness^c^ (hours)	51, 56, 14–84
Time spent on paid work per week at 9 months post-discharge^c^ (hours)	40.5, 45, 14–7
Time spent on unpaid work per week before illness (hours)	10, 4.5, 0–63
Time spent on unpaid work per week at 9 months post-discharge (hours)	15.6, 14, 0–56
Impact of diseases on income ($US) (mean, median, minimum–maximum)	
Patient’s monthly income before illness^c^	278, 139, 9–1484
Income loss due to hospitalization^d^	173, 139, 0–696
Patient’s monthly income at 9 months post-discharge^c^	237, 209, 28–742
Per capita family monthly income before illness^c^	79, 70, 5–209
Per capita family monthly income at 9 months post-discharge^c^	83, 79, 9–241

^a^Chi-square test for difference in probability of returning to paid work by 9 months between those with severe hearing loss and non-severe hearing loss had a p-value of 0.09.

^b^Among patients who returned to paid work by 9 months after hospital discharge.

^c^Calculated among patients in paid work only.

^d^Includes both income loss of the patient and caregivers.

The disease was reported to greatly impact patients and their family in most cases (43/45 patients at discharge and 20/46 after 9 months) (see Supplementary Table 2). Reduced productivity in paid work was the leading factor causing the negative impact, followed by an increase in health-related expenses. Many families resorted to taking out loans to cover hospital costs (33/45 at discharge) and 9/31 families continued to pay loan interest 9 months after discharge (see Supplementary Table 3).

## Discussion

This study aimed to quantify the burden of human illness caused by *S. suis* infection in Viet Nam and showed a significant impact on affected patients and their families in this low- and middle-income country (LMIC). Most patients were the main family income generator and had non-skilled occupations, including household farming and manual labour. The affected families had to pay a higher hospital cost than their annual per capita family income. This cost was also higher than the average annual per capita income of the general population (31 680 000 VND or US$1471).^19^

*S. suis* infection created significant catastrophic health expenditures for the families, aggravated by the significant loss in productivity due to long-term hearing loss and dizziness. Families affected by illness often needed to make decisions about treatment and manage their financial resources to pay for the treatment and replace the lost income.^20^ This was particularly the case for families who did not have any savings and for whom farming was their primary work for subsistence. Studies have shown that families in LMICs depend on their established social networks for their coping capacity, and the poorest families often have the weakest support network for finding financial resources they need in these circumstances.^20–22^ Poor families are often forced to borrow money from high-interest sources, as was reported by many families in our study.

We utilized a combination of data sources, including surveillance data, retrospective hospital data and prospective patient follow-up data, and used a statistically principled method of multiple imputation to estimate the true values for incidence rates based on reported surveillance data. We assumed that incidence data for the provinces that had a report of zero cases were likely missing data and these values could be estimated based on a set of relevant geographic, demographic and socio-economic indicators for which data were available. Even when data were imputed for potentially missing data in the surveillance system, our estimate of 0.25–0.32 *S. suis* cases per 100 000 population in Viet Nam in the years 2011–2014 is likely a conservative estimate of incidence. A previous prospective study provided a slightly higher estimate of 0.57 cases per 100 000 person-years (95% CI 0.47 to 0.70) in the adult population during 2007–2010,^23^ equivalent to 0.422 cases per 100 000 person-years for the whole population. However, this study was only conducted in 12 hospitals in the southern and central parts of Viet Nam, which may be an overrepresentation of the areas with high disease incidence.

We used an incidence-based approach to quantify the burden of *S. suis* infection. This approach is appropriate for studying the health burden of infectious diseases with long-term sequelae and has been used previously for enteric pathogens.^9,24^ Using a prevalence-based approach, it would have been more difficult to include the impact of hearing loss with and without vestibular dysfunction, because calculating the prevalence of such sensory problems attributable to a specific cause is impossible in the context of Viet Nam. Long-term consequences of infectious diseases are often neglected in the assessments of disease burden.^25,26^ In the analysis of national burden of disease and injury for Viet Nam in 2008, only 13% of the total burden was attributed to infectious diseases and neonatal and maternal conditions, of which >75% were caused by mortality due to such communicable conditions.^27^ This implies a lack of data for long-term morbidity of infectious diseases in this country.

We reported a higher cost of hospitalization for *S. suis* infection than the costs documented in previous studies for meningitis in children in Viet Nam (US$44 per suspected case, US$164 per definite case in a provincial hospital in 2006^28^ and US$300 per case in a central hospital in 2012).^29^ Comparison of the costs across different studies has limitations due to different frames of cost analysis, year of estimation and level of health care settings. However, the higher hospital costs found in our study are likely to reflect the disease severity and sequelae of *S. suis* infection.

Very few studies in LMICs have calculated the indirect costs due to illness. Here we estimated income and productivity loss due to hospitalization, long-term sequelae and premature mortality from *S. suis* infection. Our estimation for indirect costs was based on the human capital approach, which has been criticized for overestimating the economic impact of disease compared with the friction cost approach, which only counts the production loss while finding a replacement worker.^30^ However, the latter approach was not suitable for estimating the cost of illness to the affected households, as the majority of *S. suis* patients are in informal employment (such as household farming). Our cost estimates were sensitive to a number of input parameters, especially disease-related factors, which reflects the wide clinical spectrum and degree of severity of this infection in humans. The variation in the annual average national income from 2011 to 2014 also affected our cost estimates, however, this impact is likely to represent the actual economic fluctuations in the fast-changing market economy of the country. The cost estimates were also influenced by the level of productivity reduction in hearing-impaired individuals and further studies are warranted to investigate this impact in an LMIC such as Viet Nam.

Findings from our estimation of health and economic burden should be interpreted in light of potential limitations. First, our direct cost estimates were based on data collected at a referral hospital, which may not be representative for cases of *S. suis* infection treated at lower-level hospitals. The median length of stay for our patients was 23 d (range 9–56), which is higher than the average of 17 d (range 0–77) reported in the literature,^2^ although still within the same range. Second, we did not include long-term audiologic assessments and fitting of assistive devices in the direct medical costs or special education and rehabilitation, including vocational training, for hearing impaired people in the indirect costs. These could not be captured during the follow-up period of the study.

In conclusion, this study demonstrates a considerable burden of disease and economic impact of *S. suis* infection on patients and families in an LMIC like Viet Nam. The high burden was beyond the acute infection and associated hospitalizations and encompassed the long-term consequences caused by premature mortality and disabling sequelae, especially when most of the infected individuals were the main breadwinners of their families. This work highlights the importance of improving disease monitoring and surveillance to provide accurate data for public health planning and interventions. It also provides consolidating evidence for strengthening disease prevention activities in the country.

## Supplementary Material

Supplementary Data
