# Supplementary material for: Burden of disease and economic impact of human Streptococcus suis infection in Viet Nam
Source: Trans R Soc Trop Med Hyg. 2019 Feb 27;113(6):341–50. doi: 10.1093/trstmh/trz004 (PMC6580695; doi:10.1093/trstmh/trz004)
Supplement: Supplementary Data [file trz004_supplementary_data.docx]

Supplementary Data

Manuscript title: Burden of disease and economic impact of human *Streptococcus suis* infection in Viet Nam

Supplementary Methods

Multiple imputation for estimating disease incidence

This process was done using the MICE (multiple imputation by chained equations) package in the R software.^1^ We applied a predictive mean matching procedure which imputed missing values using nearest-neighbor donors based on the distance estimated from the expected values conditional on observed variables. This method often yields plausible estimates while maintaining underlying data distribution and is widely used for semicontinuous skewed data.^2^ To assess convergence of the imputation process, the mean and variance for each imputed dataset (sequence) were plotted against the iterations. Convergence was reached when the sequences did not show any definite trends and the variance between sequences was not larger than the variance within the sequences. We also checked the distributions of the residuals of the missing data model for observed data and imputed data against the propensity score for missing probability. Overlap in these two distributions indicates an appropriate imputation process.

DALY calculations

The Disability Adjusted Life Year (DALY) measure, as used in the Global Burden of Disease and injury (GBD) project,^3^ is the summation of the number of years of life lost due to premature mortality (YLL) and the number of years of healthy life lost due to disability (YLD). YLL and YLD are calculated using the following formula:

$YLD= \sum n_{l}^{a,s}*t_{l}^{a,s}*w_{l}^{a,s}$ and $YLL= \sum d_{l}^{a,s}*e_{l}^{a,s}$,

where *n* is the number of incident cases, *t* the duration, and *w* the disability weight of a specific health state or outcome (*l*) at the age of infection *a* and for sex *s*; and *d* is the number of fatal cases, *e* the remaining life expectancy at the age of death *a* and for sex *s*.

Disability weights were obtained from the recent Global Burden of Disease report for the year 2013.^4^ Weights for the state of having both hearing loss and vestibular dysfunction was derived using a multiplicative method^5,6^ combining specific disability weights (dw) for hearing loss (hl) and vestibular dysfunction (vd) based on the following formula: dw_combined_ = 1 - (1-dw_hl_) x (1-dw_vd_) . The value of vertigo was used to describe vestibular dysfunction, which was 0.113 (95%UI: 0.074-0.158). The disability weight value for profound level was used for the combined profound/ complete group.

We estimated DALY using the DALY Calculator - a Graphical User Interface for stochastic calculation of DALY developed in the R environment.^7^ Estimates were for the following age groups by sex: 15-44, 45-59 and ≥60 (*S. suis* infection has not been reported in children in Viet Nam). Uncertainty was determined through Monte Carlo simulations by iteratively simulating random values for each parameter according to its predefined distribution. Sensitivity analysis was performed using linear regression of the overall DALY estimates against the simulated values for the input parameters to identify parameters that cause significant uncertainty in the overall DALY estimates.

Cost of illness

This cost of illness approach is frequently used to establish the costs for illness and diseases by identifying all associated cost-generating components and attributing a monetary value to them.^8^

The average direct cost per one hospitalization (*Q*) was calculated by summing the average direct medical cost (formal fees and informal hospital fees, costs of drugs and other medical goods purchased outside the hospital) and the average direct non-medical cost per patient, and did not include the health system costs. The annual direct cost of human *S. suis* infection for the whole country is the product of the annual number of *S. suis* cases (*n*) and the average direct cost for treating one episode of *S. suis* infection (*Q*): $DC=n \times Q$

The indirect cost is a summation of patient’s productivity losses due to premature mortality (*IC_mortality_*), morbidity from long-term sequelae *(IC_morbidity_*), and morbidity from short-term acute infection and post-discharge recovery (*IC_acute_*). The estimation for indirect costs due to premature deaths and long-term sequelae is based on the human capital approach which takes a patient’s perspective and considers productivity loss as the product of time loss and average potential earnings.^9^ Short-term indirect costs were estimated based on reported income loss by patients and their caregivers in our follow-up study.^10^

These indirect cost components were calculated as follows:

${IC}_{mortality}=CFR \times\sum(n^{a,s}\times{PVLE}^{a,s})$,

where $n^{a,s}$is the number of *S. suis* cases per year by sex (*s*) and 5-year age group (*a*) and *CFR* is average case fatality rate of *S. suis* infection.^11^ ${PVLE}^{a,s}$is the present value of lifetime earnings for each sex and age group, taking into account life expectancy, average national income per capita and the proportions of individuals potentially active in the workforce based on the human capital approach^12^ and adapted for the Vietnamese context^13^ (see details on calculation of PVLE below).

${IC}_{morbidity}=V \times HLR \times\sum{(n}_{sa}\times{PVLE}_{sa})$,

where *HLR* is the rate of patients with severe to complete hearing loss among those who had *S. suis* infection,^10^ and *V* is the level of earning reduction for patients survived with hearing loss compared with a normal person. Hearing loss is the main sequela in patients surviving *S. suis* infection, with or without vestibular involvement, and tends to persist over a long time period. *V* is fixed at 40% based on the estimates calculated for people with severe to profound hearing loss in the US.^14^

${IC}_{acute}= \sum(n^{a,s}*P)$,

where *P* is the average income loss per episode of hospitalization due to lost productive time spent for treatment, which is estimated from our follow-up study.^10^

PVLE Calculations

Present Value of Lifetime Earnings (PVLE) is calculated following the methodology described by Max and colleagues^12^ with the formula below.

$${PVLE}_{y,g}=\sum_{n=y}^{85+} P_{y,g}\left( n \right)\left[ Y_{g}\left( n \right)E_{g}\left( n \right)+Y_{g}^{h}\left( n \right)E_{g}^{h}\left( n \right) \right]*\left( 1+p \right)^{n-y}/{(1+r)}^{n-y}$$

where,

${PVLE}_{y,g}$ is the present discounted value of lifetime earnings for a person of age y and gender g;

$P_{y,g}\left( n \right)$is the probability that a person of age y and gender g will survive to age n;

*y* is the age of the person at present;

*g* is the gender of the person;

*n* is the age of the person;

$Y_{g}\left( n \right)$ is the mean annual earnings of an employed person of gender g and age n;

$E_{g}\left( n \right)$ is the proportion of the population of gender g and age n that are employed in the labor market;

$Y_{g}^{h}$*(n)* is the mean annual imputed value of household production for a person of gender g and age n;

$E_{g}^{h}\left( n \right)$ is the proportion of the population of gender g and age n that are keeping house;

*p* is the rate of increase of labor productivity;

*r* is the real discount rate.

This approach values the productivity of a life based on market earnings and imputed values for housekeeping services. In our study, we did not include the value of household and unpaid work due to a lack of data on the wage for housekeeping services and measurement of time and frequency spent on these activities in settings such as Viet Nam.

The values and (or) sources of each indicator which were used for calculation are as follows:

- Probability of death and survival: obtained from Viet Nam Lifetable 2009 (General Statistics Office Viet Nam)
- Annual earnings: based on average household income per capita reported in National Statistical Yearbooks 2011-2014
- Proportion of population employed in labor market: obtained from reports of work and labor surveys published by General Statistics Office Vietnam 2011-2014
- Rate of increase of labor productivity: averaged growth rate of GDP from 2011-2014, fixed at 6%
- Discount rate: 3%

Supplementary Tables

Supplementary Table 1. Predictor variables in the imputation model for disease incidence in 2011-2014

| **Code** | **Province** | **Region** | **Population density**  **(persons/ km2)** | | | | **Pig density**  **(pigs/km2)** | | | | **% rural population** | | | | **No. hospitals** | | | | **Monthly**  **income**  **(1000VND**  **/person)** | **Immigration rate**  **(per 1000 population)** | | | | **Outmigration rate**  **(per 1000 population)** | | | | **% skilled workers** | | | |
| --- | --- | --- | --- | --- | --- | --- | --- | --- | --- | --- | --- | --- | --- | --- | --- | --- | --- | --- | --- | --- | --- | --- | --- | --- | --- | --- | --- | --- | --- | --- | --- |
|  |  |  | 2011 | 2012 | 2013 | 2014 | 2011 | 2012 | 2013 | 2014 | 2011 | 2012 | 2013 | 2014 | 2011 | 2012 | 2013 | 2014 |  | 2011 | 2012 | 2013 | 2014 | 2011 | 2012 | 2013 | 2014 | 2011 | 2012 | 2013 | 2014 |
| 1 | An Giang | 8 | 608 | 609 | 609 | 610 | 50.3 | 48.2 | 42.8 | 29.7 | 68.7% | 70.0% | 69.8% | 69.7% | 15 | 15 | 15 | 4.3 | 1871.5 | 3.9 | 2.2 | 3.1 | 4.3 | 16 | 11.2 | 11.3 | 12.7 | 8.0% | 8.9% | 10.6% | 9.8% |
| 2 | Ba Ria Vung Tau | 7 | 516 | 522 | 529 | 533 | 152.4 | 151.4 | 151.6 | 154.5 | 50.1% | 50.1% | 50.2% | 49.5% | 10 | 10 | 12 | 15 | 2903.8 | 16.5 | 10.5 | 10.8 | 15 | 10.8 | 7.7 | 6.9 | 7.8 | 16.1% | 21.3% | 22.5% | 24.0% |
| 3 | Bac Giang | 2 | 410 | 413 | 414 | 422 | 303.9 | 304.8 | 310.1 | 315.5 | 90.1% | 90.2% | 90.3% | 88.7% | 16 | 15 | 16 | 4.8 | 1567.8 | 5.5 | 3.2 | 4.6 | 4.8 | 10.4 | 6.8 | 9.2 | 5.8 | 13.9% | 13.8% | 15.5% | 15.6% |
| 4 | Bac Kan | 2 | 61 | 62 | 62 | 63 | 37.8 | 36.8 | 37.3 | 37.6 | 83.8% | 83.7% | 83.8% | 83.5% | 9 | 9 | 9 | 4 | 1141.9 | 4.8 | 3.1 | 4.7 | 4 | 6.7 | 4.6 | 11.7 | 10.1 | 13.8% | 11.6% | 11.9% | 11.9% |
| 5 | Bac Lieu | 8 | 354 | 354 | 355 | 356 | 91.9 | 89.6 | 85.1 | 87.2 | 73.1% | 72.7% | 72.7% | 73.6% | 7 | 6 | 7 | 2.5 | 2035.4 | 3 | 2.4 | 2.5 | 2.5 | 9.6 | 10.7 | 16.4 | 16.6 | 6.5% | 8.6% | 9.7% | 9.6% |
| 6 | Bac Ninh | 1 | 1289 | 1313 | 1354 | 1375 | 476.6 | 468.0 | 456.3 | 466.4 | 74.0% | 73.8% | 73.6% | 72.5% | 11 | 12 | 12 | 15.4 | 2501.8 | 13.4 | 12.7 | 19.4 | 15.4 | 6.5 | 5.5 | 6.1 | 5.4 | 18.9% | 19.3% | 20.4% | 21.1% |
| 7 | Ben Tre | 8 | 533 | 534 | 535 | 535 | 189.1 | 182.8 | 182.7 | 190.8 | 90.0% | 90.0% | 90.0% | 89.8% | 12 | 12 | 12 | 7.4 | 1579.8 | 5.8 | 2.5 | 5.3 | 7.4 | 15.5 | 10.8 | 9.2 | 13.9 | 9.3% | 7.4% | 8.5% | 9.4% |
| 8 | Binh Dinh | 5 | 247 | 248 | 250 | 250 | 109.1 | 107.5 | 110.1 | 115.8 | 72.3% | 69.2% | 69.2% | 69.0% | 17 | 17 | 18 | 4.4 | 1719 | 6.6 | 2.8 | 3.7 | 4.4 | 10 | 9.8 | 7.5 | 8.2 | 11.6% | 12.6% | 15.2% | 14.1% |
| 9 | Binh Duong | 7 | 628 | 649 | 669 | 695 | 166.0 | 164.5 | 165.4 | 171.4 | 35.9% | 35.2% | 35.5% | 23.2% | 10 | 8 | 9 | 70.2 | 3567.8 | 64.8 | 59.1 | 54.5 | 70.2 | 22.1 | 10.2 | 19.9 | 18.2 | 15.0% | 14.3% | 17.0% | 18.1% |
| 10 | Binh Phuoc | 7 | 132 | 133 | 134 | 136 | 29.3 | 29.7 | 30.6 | 31.4 | 83.2% | 83.2% | 83.2% | 80.7% | 12 | 12 | 12 | 8 | 2217.6 | 10.9 | 6.7 | 7.1 | 8 | 11.7 | 8.9 | 9.2 | 12 | 12.3% | 13.0% | 14.5% | 15.7% |
| 11 | Binh Thuan | 5 | 151 | 153 | 154 | 155 | 26.3 | 26.4 | 26.8 | 30.6 | 60.7% | 60.7% | 60.7% | 60.7% | 13 | 13 | 13 | 2.4 | 1746.6 | 2.1 | 2.3 | 5.3 | 2.4 | 8.5 | 6.1 | 8.5 | 6.9 | 9.1% | 10.6% | 10.5% | 12.0% |
| 12 | Ca Mau | 8 | 229 | 230 | 230 | 230 | 42.6 | 40.3 | 36.3 | 25.2 | 78.5% | 78.4% | 78.4% | 77.4% | 12 | 12 | 13 | 2.8 | 1778.8 | 4.2 | 2.4 | 2.9 | 2.8 | 16.3 | 8.2 | 11.4 | 16.4 | 5.5% | 5.0% | 7.5% | 7.1% |
| 13 | Can Tho | 8 | 852 | 862 | 868 | 879 | 89.6 | 88.9 | 76.6 | 79.8 | 34.0% | 33.7% | 33.5% | 33.3% | 17 | 16 | 17 | 8.2 | 2324.9 | 6.9 | 8.9 | 9.6 | 8.2 | 11 | 7.1 | 13.6 | 11.4 | 13.0% | 14.7% | 15.2% | 16.9% |
| 14 | Cao Bang | 2 | 77 | 77 | 77 | 78 | 52.8 | 53.1 | 56.2 | 56.6 | 83.0% | 79.9% | 78.6% | 76.9% | 16 | 16 | 17 | 3.3 | 1053.5 | 4.6 | 4.4 | 8.1 | 3.3 | 10.1 | 15.6 | 8.6 | 8.2 | 15.0% | 19.1% | 19.3% | 20.0% |
| 15 | Da Nang | 5 | 740 | 758 | 772 | 784 | 45.6 | 43.8 | 44.6 | 45.9 | 12.9% | 12.8% | 12.7% | 12.7% | 12 | 13 | 14 | 14 | 2865.2 | 22.8 | 17.5 | 15.5 | 14 | 7.9 | 6.3 | 9.7 | 13.8 | 33.2% | 34.8% | 35.9% | 37.5% |
| 16 | Dac Nong | 6 | 79 | 83 | 85 | 88 | 23.0 | 22.3 | 21.7 | 20.6 | 84.8% | 84.6% | 84.8% | 84.8% | 8 | 8 | 8 | 42.7 | 1610.8 | 5.7 | 42.3 | 18.4 | 42.7 | 10.7 | 6.1 | 6.7 | 7.1 | 7.2% | 8.4% | 9.4% | 8.0% |
| 17 | Daklak | 6 | 135 | 137 | 139 | 140 | 53.7 | 53.4 | 55.0 | 55.2 | 76.0% | 75.9% | 75.9% | 75.7% | 20 | 20 | 20 | 3.7 | 1639.2 | 7.7 | 8.4 | 9.7 | 3.7 | 12.3 | 7.8 | 8.5 | 6.1 | 10.4% | 12.5% | 14.5% | 13.2% |
| 18 | Dien Bien | 3 | 54 | 54 | 55 | 56 | 30.3 | 30.2 | 30.9 | 31.7 | 85.0% | 85.0% | 85.0% | 84.9% | 12 | 13 | 14 | 4.4 | 819.4 | 3.9 | 3.4 | 4.7 | 4.4 | 2.1 | 3.6 | 4.5 | 2.7 | 13.2% | 17.1% | 18.9% | 19.9% |
| 19 | Dong Nai | 7 | 451 | 461 | 469 | 481 | 225.0 | 221.2 | 223.5 | 240.2 | 66.3% | 66.1% | 65.8% | 65.5% | 17 | 16 | 16 | 23.6 | 2576.7 | 31.4 | 18.5 | 14.6 | 23.6 | 9.3 | 6 | 7.9 | 11.7 | 12.3% | 13.9% | 16.1% | 15.0% |
| 20 | Dong Thap | 8 | 495 | 496 | 497 | 498 | 81.2 | 81.3 | 74.8 | 66.9 | 82.2% | 82.2% | 82.2% | 82.1% | 16 | 16 | 16 | 3.2 | 1665.5 | 5.8 | 2.5 | 3.8 | 3.2 | 11.1 | 8.8 | 9.5 | 9.6 | 7.4% | 7.0% | 9.1% | 8.7% |
| 21 | Gia Lai | 6 | 85 | 86 | 88 | 89 | 25.2 | 25.1 | 25.8 | 26.2 | 69.8% | 70.6% | 70.6% | 70.5% | 20 | 19 | 21 | 5.2 | 1563.5 | 4.9 | 4.9 | 8 | 5.2 | 6.9 | 5.3 | 8.1 | 7.1 | 9.3% | 10.5% | 10.4% | 10.8% |
| 22 | Ha Giang | 2 | 94 | 96 | 97 | 100 | 58.2 | 56.8 | 55.0 | 58.2 | 85.0% | 84.9% | 85.0% | 85.0% | 15 | 14 | 14 | 1.4 | 850.3 | 2.4 | 1.3 | 2.8 | 1.4 | 3.6 | 1.7 | 2.6 | 2.1 | 10.8% | 9.8% | 9.0% | 9.6% |
| 23 | Ha Nam | 1 | 914 | 918 | 923 | 927 | 864.7 | 406.5 | 409.8 | 430.5 | 89.5% | 89.5% | 89.4% | 84.5% | 12 | 12 | 12 | 6.7 | 1753.9 | 6.6 | 1.4 | 6.1 | 6.7 | 10.5 | 5.6 | 6.2 | 8.7 | 14.9% | 15.8% | 16.1% | 16.3% |
| 24 | Ha Noi | 1 | 2013 | 2059 | 2087 | 2134 | 460.5 | 414.3 | 415.2 | 427.3 | 56.8% | 57.2% | 57.5% | 55.5% | 40 | 41 | 41 | 7.5 | 2944.9 | 11 | 6.1 | 7.7 | 7.5 | 6.4 | 3.3 | 7.4 | 7.8 | 30.6% | 35.3% | 36.2% | 38.4% |
| 25 | Ha Tinh | 4 | 205 | 205 | 207 | 209 | 55.6 | 54.7 | 56.2 | 59.9 | 84.0% | 84.4% | 84.5% | 84.4% | 17 | 16 | 17 | 10.9 | 1298.9 | 6.2 | 3.7 | 6.9 | 10.9 | 12.4 | 12.8 | 11 | 14.9 | 12.2% | 17.7% | 19.0% | 20.4% |
| 26 | Hai Duong | 1 | 1038 | 1048 | 1055 | 1065 | 324.6 | 324.3 | 328.7 | 336.5 | 78.1% | 78.0% | 77.9% | 76.9% | 21 | 19 | 20 | 4 | 2047 | 7.8 | 5.6 | 5.6 | 4 | 5.5 | 4.8 | 8.5 | 5.6 | 13.4% | 14.7% | 13.4% | 15.0% |
| 27 | Hai Phong | 1 | 1233 | 1250 | 1260 | 1274 | 355.4 | 345.8 | 337.3 | 319.0 | 53.6% | 53.5% | 53.4% | 53.3% | 24 | 23 | 24 | 4 | 2526.2 | 7.1 | 6 | 6.6 | 4 | 5 | 2.1 | 4 | 3.4 | 23.6% | 24.0% | 26.6% | 27.6% |
| 28 | Hau Giang | 8 | 480 | 480 | 483 | 480 | 73.4 | 72.1 | 72.1 | 74.0 | 77.1% | 73.7% | 76.2% | 75.8% | 8 | 9 | 9 | 4.5 | 1527.4 | 7.7 | 7.7 | 6.7 | 4.5 | 5.4 | 11.6 | 10.8 | 18 | 8.2% | 8.6% | 8.6% | 9.7% |
| 29 | HCMC | 7 | 3589 | 3666 | 3731 | 3809 | 147.8 | 149.1 | 136.8 | 138.9 | 16.9% | 16.9% | 17.5% | 17.9% | 52 | 50 | 52 | 16.9 | 3652.7 | 25 | 14.8 | 16.5 | 16.9 | 13.5 | 7.2 | 10.3 | 11.4 | 29.3% | 28.4% | 31.6% | 32.5% |
| 30 | Hoa Binh | 3 | 174 | 175 | 175 | 177 | 94.5 | 92.5 | 83.4 | 85.5 | 85.0% | 85.0% | 84.9% | 85.5% | 14 | 14 | 14 | 3.4 | 1219.2 | 5.6 | 2.1 | 2.7 | 3.4 | 7.8 | 5.8 | 10.9 | 8.4 | 15.8% | 16.7% | 17.9% | 15.9% |
| 31 | Hung Yen | 1 | 1242 | 1237 | 1244 | 1251 | 696.1 | 691.5 | 673.1 | 636.3 | 87.4% | 87.0% | 86.7% | 86.9% | 17 | 17 | 17 | 7.4 | 1803.2 | 8 | 7 | 10.9 | 7.4 | 6.1 | 9.6 | 6.5 | 5.2 | 13.2% | 16.8% | 17.7% | 20.0% |
| 32 | Khanh Hoa | 5 | 225 | 227 | 229 | 229 | 20.8 | 21.1 | 21.7 | 24.4 | 50.2% | 55.5% | 55.5% | 55.2% | 13 | 13 | 13 | 3.5 | 1896.1 | 5 | 3.1 | 3.4 | 3.5 | 8.4 | 5.5 | 4.7 | 7.4 | 14.1% | 13.7% | 13.6% | 16.1% |
| 33 | Kien Giang | 8 | 270 | 272 | 274 | 275 | 51.6 | 50.8 | 51.5 | 52.7 | 72.8% | 72.7% | 72.7% | 73.4% | 13 | 13 | 13 | 3 | 1962.8 | 7.3 | 4.4 | 4.1 | 3 | 15.2 | 8.8 | 10.8 | 9.6 | 9.9% | 9.0% | 9.7% | 9.3% |
| 34 | Kon Tum | 6 | 47 | 48 | 49 | 50 | 12.4 | 12.6 | 12.8 | 12.9 | 65.5% | 65.0% | 64.5% | 64.7% | 9 | 10 | 11 | 6.7 | 1294.4 | 8.8 | 11.8 | 13.3 | 6.7 | 9.8 | 5.3 | 5.9 | 7.6 | 15.3% | 13.1% | 12.8% | 12.4% |
| 35 | Lai Chau | 3 | 43 | 44 | 45 | 46 | 22.5 | 20.0 | 19.9 | 19.8 | 85.7% | 85.6% | 85.5% | 82.8% | 10 | 10 | 11 | 3.8 | 758 | 3.5 | 3.9 | 5 | 3.8 | 4.1 | 3.4 | 4.7 | 3.8 | 10.9% | 11.3% | 11.8% | 13.5% |
| 36 | Lam Dong | 6 | 125 | 126 | 128 | 129 | 35.3 | 35.3 | 34.9 | 35.9 | 61.9% | 61.8% | 61.7% | 61.0% | 14 | 14 | 14 | 6 | 1848.4 | 9.2 | 6.8 | 8 | 6 | 8.3 | 8.9 | 8.4 | 9.1 | 13.0% | 14.5% | 16.3% | 14.7% |
| 37 | Lang Son | 2 | 89 | 89 | 90 | 91 | 40.0 | 39.5 | 40.4 | 39.6 | 80.8% | 80.8% | 80.7% | 80.5% | 14 | 13 | 14 | 2 | 1212.4 | 6.2 | 3.5 | 6.1 | 2 | 8.1 | 7.8 | 6.3 | 7.1 | 12.4% | 13.6% | 16.4% | 15.8% |
| 38 | Lao Cai | 3 | 100 | 101 | 103 | 104 | 66.2 | 64.7 | 65.7 | 68.0 | 78.7% | 77.4% | 77.3% | 77.1% | 13 | 12 | 12 | 2 | 1085.1 | 3.4 | 2.9 | 4.4 | 2 | 2.4 | 3 | 5.1 | 4.1 | 13.2% | 14.1% | 16.2% | 15.6% |
| 39 | Long An | 8 | 323 | 325 | 327 | 329 | 59.4 | 56.5 | 56.4 | 57.6 | 82.2% | 82.0% | 82.0% | 82.0% | 16 | 16 | 21 | 6.2 | 1956.3 | 3.8 | 4.7 | 4.7 | 6.2 | 7.7 | 7.5 | 6.8 | 8.8 | 8.5% | 9.5% | 11.4% | 10.9% |
| 40 | Nam Dinh | 1 | 1110 | 1112 | 1113 | 1116 | 677.2 | 449.9 | 444.3 | 467.9 | 82.0% | 81.9% | 81.9% | 81.8% | 18 | 18 | 18 | 4.6 | 1791.1 | 6.1 | 2.3 | 3.5 | 4.6 | 7.4 | 7.8 | 8.4 | 8.3 | 11.7% | 13.3% | 15.7% | 11.0% |
| 41 | Nghe An | 4 | 178 | 179 | 181 | 184 | 64.7 | 61.0 | 61.5 | 58.9 | 86.7% | 86.4% | 86.5% | 84.9% | 26 | 28 | 29 | 19.4 | 1366.6 | 4.8 | 1 | 6.8 | 19.4 | 11.8 | 10.5 | 12.5 | 10.1 | 12.1% | 14.4% | 15.0% | 15.6% |
| 42 | Ninh Binh | 1 | 652 | 665 | 673 | 679 | 282.0 | 280.2 | 255.5 | 262.8 | 79.9% | 81.0% | 80.9% | 80.3% | 14 | 13 | 14 | 7.9 | 1695.8 | 10.2 | 10.2 | 11.9 | 7.9 | 8.5 | 6.4 | 11.1 | 7.5 | 21.7% | 25.6% | 27.2% | 26.1% |
| 43 | Ninh Thuan | 5 | 169 | 172 | 175 | 176 | 17.3 | 17.2 | 17.6 | 19.1 | 63.9% | 63.9% | 63.8% | 63.8% | 6 | 7 | 7 | 4 | 1637.2 | 3.8 | 3.6 | 7.4 | 4 | 15.8 | 4.5 | 3.1 | 6.8 | 13.5% | 14.2% | 13.8% | 14.0% |
| 44 | Phu Tho | 2 | 375 | 378 | 382 | 385 | 186.4 | 186.2 | 188.7 | 220.1 | 79.1% | 81.8% | 81.5% | 81.4% | 17 | 16 | 16 | 5.2 | 1578.7 | 4.5 | 3.1 | 6.7 | 5.2 | 13.7 | 5 | 7 | 7.7 | 13.2% | 14.4% | 15.3% | 14.2% |
| 45 | Phu Yen | 5 | 172 | 173 | 175 | 175 | 20.0 | 19.7 | 19.7 | 20.1 | 76.8% | 76.8% | 76.8% | 71.2% | 12 | 13 | 13 | 3.9 | 1439.5 | 6.1 | 3.2 | 3.9 | 3.9 | 14 | 6.2 | 6.3 | 7.7 | 11.0% | 10.0% | 10.4% | 11.8% |
| 46 | Quang Binh | 4 | 106 | 106 | 107 | 108 | 43.9 | 42.9 | 42.8 | 44.0 | 84.8% | 84.8% | 84.8% | 80.5% | 8 | 8 | 8 | 5.8 | 1409.9 | 4 | 7.6 | 6.5 | 5.8 | 10.3 | 7.6 | 14.5 | 11.4 | 13.8% | 19.0% | 18.6% | 19.7% |
| 47 | Quang Nam | 5 | 137 | 139 | 140 | 141 | 50.4 | 49.8 | 46.8 | 47.5 | 80.7% | 80.9% | 80.9% | 80.7% | 24 | 24 | 26 | 4.6 | 1376.4 | 8.8 | 3.3 | 6.2 | 4.6 | 11.1 | 6.9 | 7.7 | 6.7 | 12.3% | 9.5% | 11.1% | 12.7% |
| 48 | Quang Ngai | 5 | 237 | 238 | 240 | 241 | 94.6 | 92.6 | 90.2 | 88.9 | 85.4% | 85.3% | 85.3% | 84.4% | 18 | 18 | 17 | 3.6 | 1300.5 | 5.4 | 1.8 | 4.2 | 3.6 | 13.3 | 7.6 | 6 | 8.4 | 9.7% | 10.6% | 12.4% | 14.1% |
| 49 | Quang Ninh | 2 | 191 | 193 | 194 | 197 | 54.2 | 54.2 | 54.4 | 55.8 | 47.9% | 47.7% | 27.5% | 38.8% | 19 | 19 | 19 | 3.8 | 2557.3 | 3.6 | 2 | 4.5 | 3.8 | 5.5 | 4.8 | 10.4 | 7.8 | 28.3% | 31.8% | 32.5% | 36.1% |
| 50 | Quang Tri | 4 | 128 | 128 | 129 | 130 | 48.3 | 47.5 | 48.3 | 50.4 | 71.2% | 70.8% | 71.0% | 70.9% | 10 | 10 | 10 | 6.2 | 1300 | 4.9 | 3.6 | 5.3 | 6.2 | 10 | 8.4 | 10.2 | 10.9 | 12.5% | 16.1% | 19.2% | 18.5% |
| 51 | Soc Trang | 8 | 394 | 393 | 395 | 395 | 84.6 | 84.3 | 84.1 | 87.7 | 74.0% | 66.0% | 71.6% | 68.1% | 12 | 11 | 11 | 3.4 | 1323.6 | 3.1 | 2 | 3.2 | 3.4 | 14.8 | 10.2 | 9.5 | 12 | 7.7% | 8.9% | 13.5% | 10.4% |
| 52 | Son La | 3 | 79 | 80 | 81 | 82 | 38.4 | 37.8 | 33.4 | 36.3 | 85.8% | 86.0% | 86.1% | 86.3% | 16 | 16 | 16 | 2 | 1019.5 | 2.7 | 1.3 | 2.6 | 2 | 1.8 | 3.4 | 2.6 | 3 | 12.3% | 12.2% | 12.0% | 13.0% |
| 53 | Tay Ninh | 7 | 268 | 270 | 272 | 274 | 52.2 | 52.7 | 48.2 | 48.3 | 84.4% | 84.3% | 84.3% | 81.3% | 12 | 12 | 12 | 5.7 | 2100.4 | 5.8 | 3.9 | 3.6 | 5.7 | 7.5 | 3.9 | 6.3 | 6.5 | 9.0% | 10.1% | 11.6% | 11.8% |
| 54 | Thai Binh | 1 | 1138 | 1138 | 1139 | 1139 | 229.4 | 700.1 | 675.9 | 655.7 | 89.7% | 90.0% | 90.0% | 90.0% | 22 | 22 | 22 | 4.7 | 1728.5 | 2.3 | 1.2 | 2.2 | 4.7 | 9.4 | 9.1 | 8.6 | 8.6 | 12.6% | 14.1% | 13.1% | 15.0% |
| 55 | Thai Nguyen | 2 | 323 | 325 | 327 | 332 | 146.3 | 145.6 | 147.2 | 154.5 | 74.0% | 71.6% | 71.5% | 69.7% | 15 | 13 | 14 | 6.5 | 1747.1 | 7.7 | 4.7 | 4.1 | 6.5 | 9.7 | 7.2 | 8.5 | 7.8 | 18.9% | 20.3% | 21.5% | 20.1% |
| 56 | Thanh Hoa | 4 | 307 | 308 | 312 | 314 | 74.6 | 69.3 | 70.9 | 75.0 | 88.9% | 88.5% | 88.1% | 85.3% | 36 | 36 | 36 | 5.7 | 1207 | 9.8 | 5.1 | 13.3 | 5.7 | 14.5 | 10.3 | 11 | 10.1 | 17.9% | 14.4% | 16.1% | 14.6% |
| 57 | Thua Thien - Hue | 4 | 219 | 221 | 223 | 225 | 46.3 | 45.7 | 43.1 | 40.1 | 48.3% | 51.8% | 51.6% | 51.4% | 13 | 14 | 16 | 7.1 | 1747.1 | 13.5 | 5 | 6.8 | 7.1 | 8.6 | 7.7 | 7.1 | 11.6 | 18.5% | 20.9% | 19.6% | 21.4% |
| 58 | Tien Giang | 8 | 671 | 675 | 679 | 684 | 225.3 | 227.8 | 224.9 | 233.2 | 84.2% | 85.3% | 85.2% | 84.6% | 11 | 11 | 11 | 7.6 | 1940.8 | 8.5 | 8.1 | 9.8 | 7.6 | 9.5 | 9.4 | 6.5 | 7.3 | 10.2% | 8.3% | 9.7% | 8.9% |
| 59 | Tra Vinh | 8 | 433 | 434 | 439 | 440 | 183.8 | 187.2 | 172.5 | 140.4 | 84.3% | 83.8% | 83.2% | 83.2% | 9 | 11 | 12 | 7.4 | 1397.9 | 11.4 | 4 | 9.5 | 7.4 | 12.7 | 8.7 | 7.3 | 12.4 | 7.8% | 12.8% | 12.3% | 13.0% |
| 60 | Tuyen Quang | 2 | 125 | 126 | 127 | 128 | 72.9 | 71.6 | 73.8 | 78.2 | 86.9% | 86.9% | 86.8% | 86.6% | 12 | 12 | 14 | 3.9 | 1162.4 | 3.7 | 2.1 | 3.3 | 3.9 | 13.5 | 3.5 | 5.1 | 6.4 | 11.0% | 16.0% | 16.5% | 18.7% |
| 61 | Vinh Long | 8 | 687 | 687 | 684 | 685 | 205.8 | 203.1 | 202.9 | 205.2 | 84.5% | 84.4% | 83.3% | 83.2% | 9 | 9 | 10 | 5.6 | 1743.9 | 7.6 | 8 | 9.9 | 5.6 | 11.5 | 9.3 | 7.2 | 10.6 | 9.1% | 10.9% | 9.9% | 12.0% |
| 62 | Vinh Phuc | 1 | 821 | 825 | 831 | 842 | 402.7 | 388.3 | 394.5 | 411.7 | 77.0% | 76.9% | 76.3% | 76.7% | 14 | 14 | 15 | 4.9 | 1866.8 | 4.3 | 2.9 | 6.4 | 4.9 | 16 | 4 | 6.8 | 4.5 | 15.1% | 19.1% | 20.5% | 20.7% |
| 63 | Yen Bai | 3 | 110 | 111 | 112 | 114 | 62.0 | 61.5 | 62.6 | 65.8 | 80.5% | 80.5% | 80.4% | 79.5% | 14 | 14 | 14 | 4.4 | 1114.3 | 4.4 | 1.8 | 4 | 4.4 | 7.4 | 4.6 | 7.4 | 5.7 | 13.2% | 12.1% | 13.7% | 13.8% |

Data sources: Statistical Yearbook of Vietnam 2011, 2012, 2013, 2014 for population density, pig population, land area, rural population, hospitals, immigration, outmigration, skilled workers; Households Living Standards Survey 2012 for income data.

Supplementary Table 2. Negative impact of *S. suis* infection on affected families rated by patients and their family members at hospital discharge and at 3 months and 9 months after hospital discharge

| Interview time-point | Great impact | Some impact | No impact |
| --- | --- | --- | --- |
| At discharge (n=45) | 43 (96%) | 2 (4%) | 0 (0%) |
| 3 months (n=31) | 18 (58%) | 6 (19%) | 7 (23%) |
| 9 months (n=46) | 20 (43%) | 11 (24%) | 15 (33%) |

Supplementary Table 3. Proportion of patients and their families reported specific negative impacts due to *S. suis* infection at different time-points

|  | Discharge  (n=45) | 3 months  (n=24) | 9 months  (n=31) |
| --- | --- | --- | --- |
| Reduced productivity in paid work | 36 (80%) | 23 (96%) | 27 (87%) |
| Reduced productivity in unpaid work | 18 (40%) | 8 (33%) | 8 (26%) |
| Increased expenses in health matters | 34 (76%) | 11 (46) | 12 (39%) |
| Cutting back on clothing | 10 (22%) | 9 (37%) | 5 (16%) |
| Cutting back on leisure/ social activities | 6 (13%) | 8 (33%) | 7 (23%) |
| Cutting back educational expenses | 2 (4%) | 1 (4%) | 2 (6%) |
| Use the savings | 16 (36%) | 0 (0%) | 1 (3%) |
| Take out loans | 33 (73%) | 9 (37%) | 9 (29%) |
| Sell capital assets | 6 (13%) | 2 (8%) | 2 (6%) |

**Supplementary Figures**

**Suplementary Figure 1. Imputed values for incidence of *S. suis* infection (mean and sd) plotted against iteration number for the imputed data by the predictive mean matching procedure. There is little trend in these streams and the streams mingle well together, indicating this was a good imputation method.**


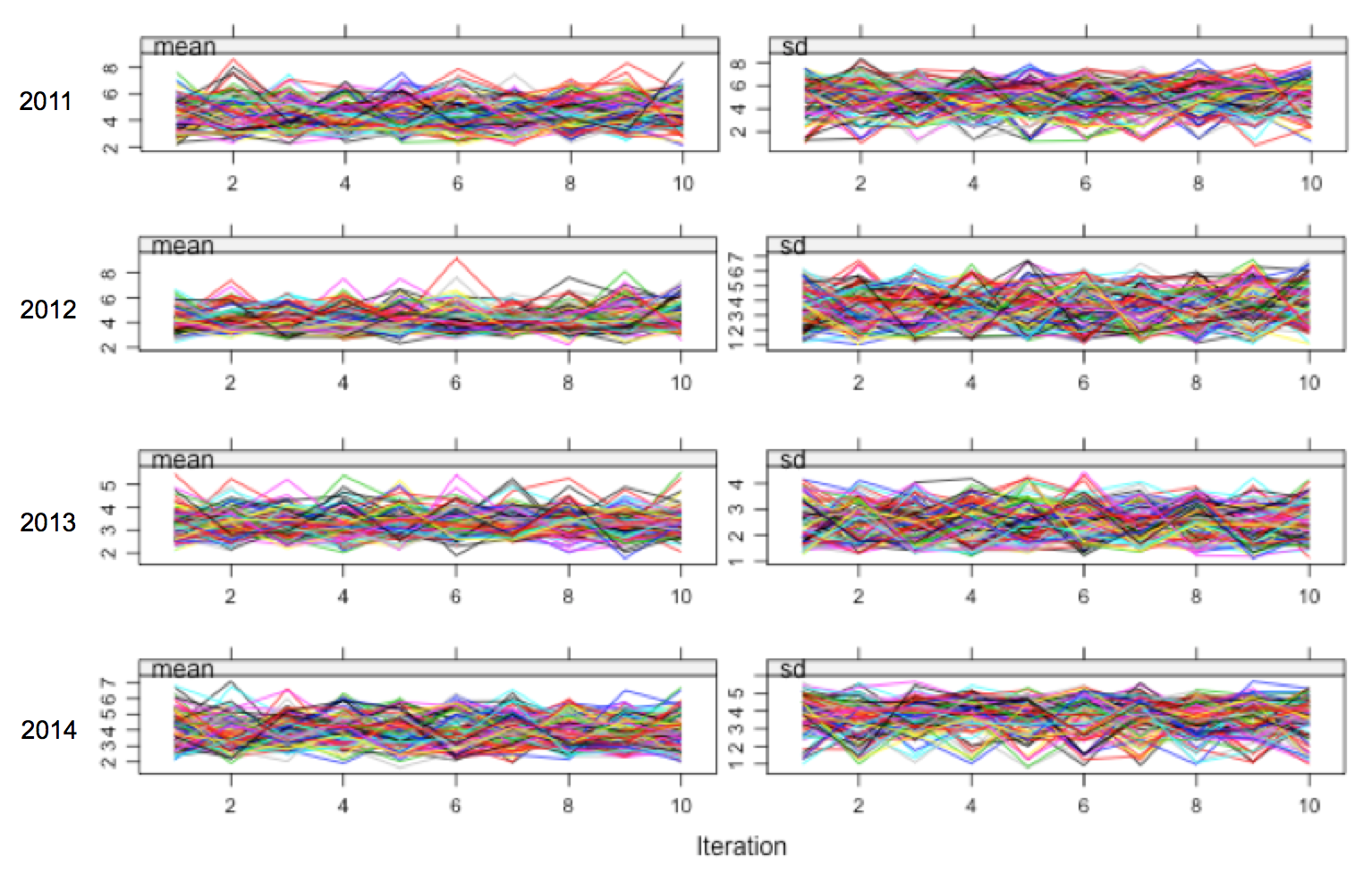


**Supplementary Figure 2. Distributions of the residuals of the missing data model using predictive mean matching (pmm) procedure for observed data (blue) and imputed data (red) conditional on pooled propensity score for missing probability. Data imputation is for *S. suis* disease incidence in 2011-2014 using all 9 predictors. Both distributions largely overlapped indicating that the overall imputation process was appropriate.**


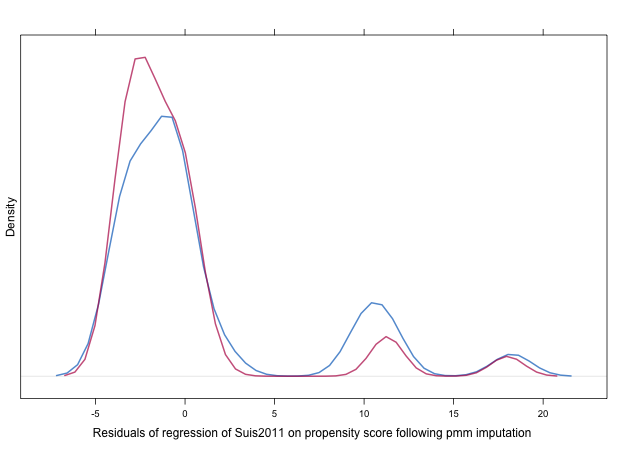


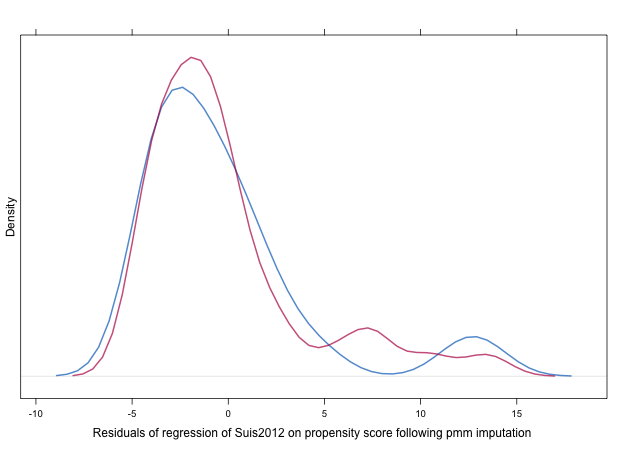


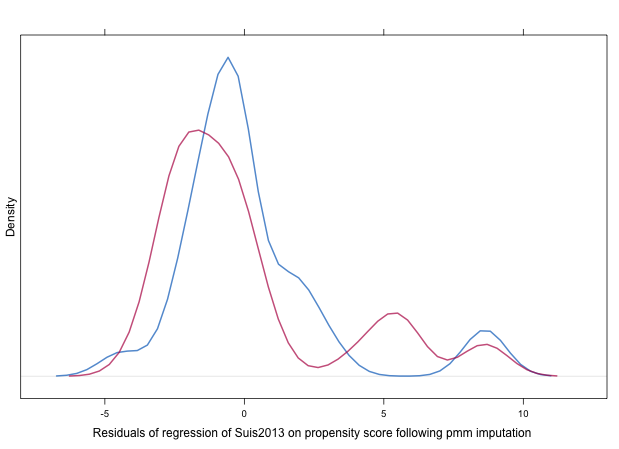


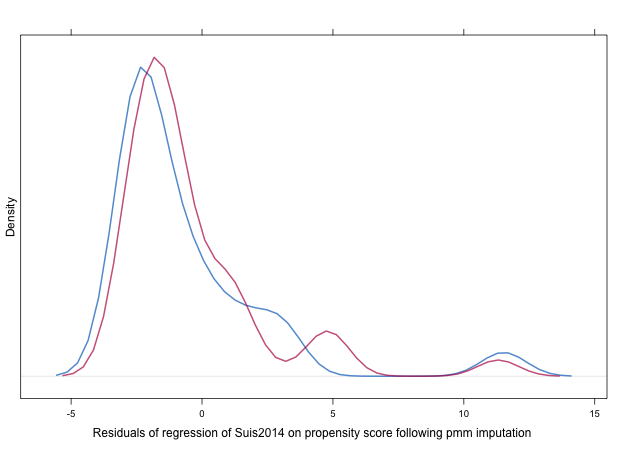


**Supplementary Figure 3. Regression-based sensitivity analysis for DALYs estimates conducted over the full range of plausible input values determined by the predefined distributions.**


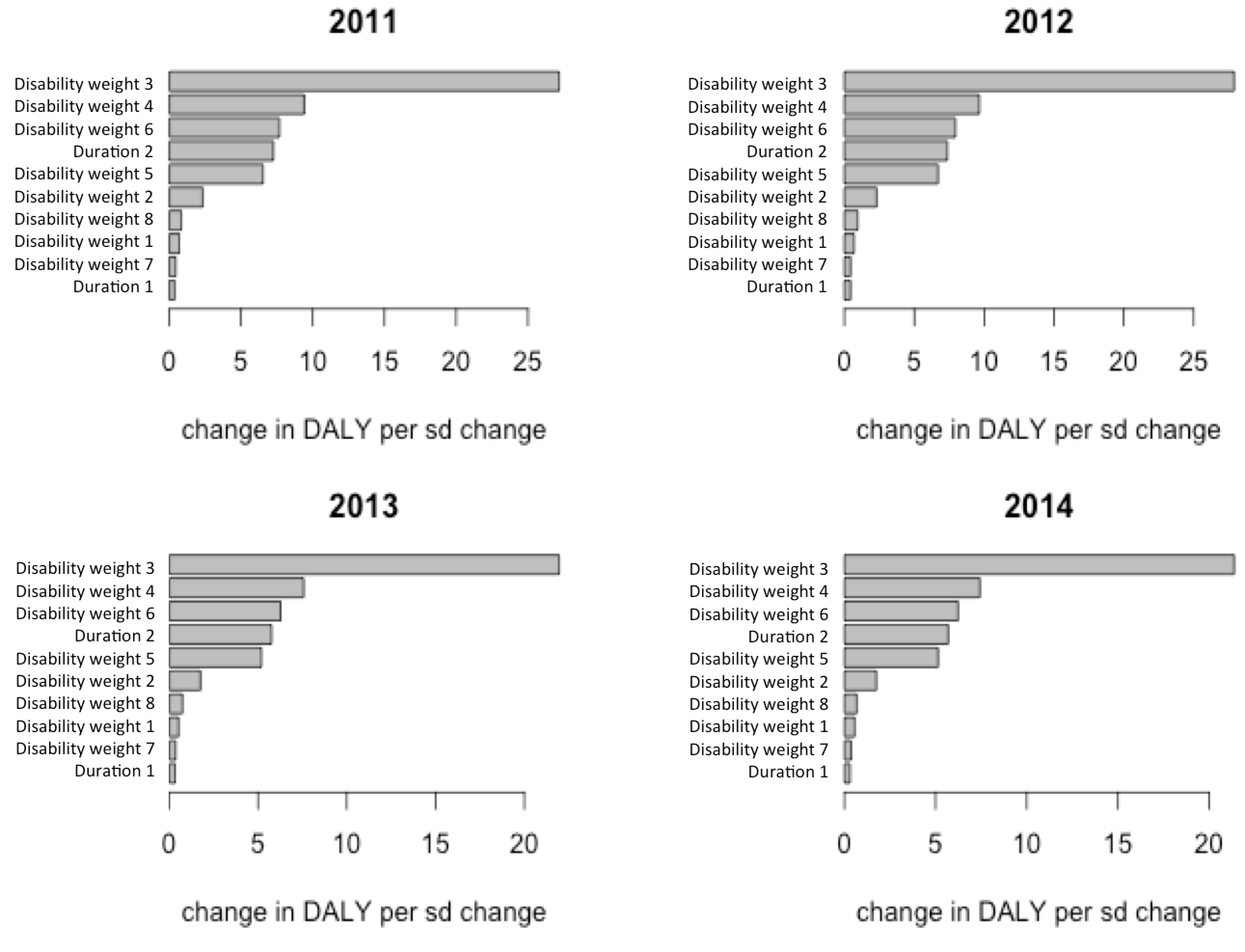


Note:

1: Acute infection;

2: Post-infectious phase;

3: Hearing loss with vestibular dysfunction/ profound to complete;

4: Hearing loss with vestibular dysfunction/ severe;

5: Hearing loss with vestibular dysfunction/ moderate;

6: Hearing loss with vestibular dysfunction/ mild;

7: Hearing loss without vestibular dysfunction/ moderate;

8: Hearing loss without vestibular dysfunction/ mild;

**Supplementary References**

1. van Buuren S, Groothuis-Oudshoorn K. MICE: Multiple Imputation by Chained Equations in R. J Stat Softw 2011;45:1-67.

2. Vink G, Frank LE, Pannekoek J, van Buuren S. Predictive mean matching imputation of semicontinuous variables. Statistica Neerlandica 2014;68:61-90.

3. Murray CJ. Quantifying the burden of disease: the technical basis for disability-adjusted life years. Bull World Health Organ 1994;72:429-45.

4. Salomon JA, Haagsma JA, Davis A, et al. Disability weights for the Global Burden of Disease 2013 study. Lancet Glob Health 2015;3:e712-23.

5. van Baal PHM, Hoeymans N, Hoogenveen RT, de Wit AG, Westert GP. Disability weights for comorbidity and their influence on Health-adjusted Life Expectancy. Popul Health Metr 2006;4:1-7.

6. Vos T, Flaxman AD, Naghavi M, et al. Years lived with disability (YLDs) for 1160 sequelae of 289 diseases and injuries 1990-2010: a systematic analysis for the Global Burden of Disease Study 2010. Lancet 2012;380:2163-96.

7. Devleesschauwer B, McDonald S, Haagsma J, et al. DALY: The DALY Calculator - A GUI for stochastic DALY calculation in R; 2014.

8. Hodgson TA, Meiners MR. Cost-of-Illness Methodology: A Guide to Current Practices and Procedures. Milbank Mem Fund Q Health Soc 1982;60:429-62.

9. van den Hout WB. The value of productivity: human-capital versus friction-cost method. Annals of the Rheumatic Diseases 2010; 69:i89-i91.

10. Huong VTL, Long HB, Kinh NV, et al. Long-term outcomes of patients with *Streptococcus suis* infection in Viet Nam: A case-control study. J Infect 2018;76(2):159-67.

11. Huong VT, Ha N, Huy NT, et al. Epidemiology, clinical manifestations, and outcomes of *Streptococcus suis* infection in humans. Emerg Infect Dis 2014;20:1105-14.

12. Max W, Rice DP, Sung HY, Michel M. Valuing human life: estimating the present value of lifetime earnings, 2000. Center for Tobacco Control Research and Education; 2004.

13. Hoang Anh PT, Thu Le T, Ross H, et al. Direct and indirect costs of smoking in Vietnam. Tob Control 2014;25:96-100.

14. Mohr PE, Feldman JJ, Dunbar JL, et al. The societal costs of severe to profound hearing loss in the United States. Int J Technol Assess Health Care 2000;16:1120-35.
